# A prospective, randomized comparison of the LMA-protector™ and i-gel™ in paralyzed, anesthetized patients

**DOI:** 10.1186/s12871-019-0785-8

**Published:** 2019-07-04

**Authors:** Jee-Eun Chang, Hyerim Kim, Jung-Man Lee, Seong-Won Min, Dongwook Won, Kwanghoon Jun, Jin-Young Hwang

**Affiliations:** 1 Department of Anesthesiology and Pain Medicine, SMG-SNU Boramae Medical Center, Boramae-ro, Dongjak-gu, Seoul, 156-707 Republic of Korea; 2 College of Medicine, Seoul National University, Seoul, Republic of Korea; 3 Department of Anesthesiology and Pain Medicine, Seoul National University Hospital, Seoul, Republic of Korea

**Keywords:** I-gel, LMA-protector™, Airway sealing

## Abstract

**Background:**

In the present study, we compare the LMA-Protector™ and the i-gel™ in terms of adequacy of the airway seal, insertion time, ease and accuracy of insertion, and the incidence of postoperative sore throat.

**Methods:**

In 110 anesthetized and paralyzed adult patients, the i-gel™ (*n* = 55) or the LMA-Protector™ (n = 55) was inserted. The primary outcome was airway leak pressure. The secondary outcomes included the first-attempt success rate, insertion time, ease and accuracy of the device insertion, ease of gastric tube placement, blood staining on the device after removal, and incidence and severity of postoperative sore throat.

**Results:**

The airway leak pressure was higher with the LMA-Protector™ than with the i-gel™ (31 [7] cmH_2_O vs. 27 [6] cmH_2_O, respectively; *P* = 0.016). Insertion time was longer with the LMA-Protector™ than with the i-gel™ (27 [16] sec vs. 19 [16] sec, respectively, *P* < 0.001), but ease of insertion and the first-attempt success rate were not different between the two groups. The LMA-Protector™ provided a worse fiberoptic view of the vocal cords and more difficult gastric tube insertion than the i-gel™ (both *P* < 0.001). Blood staining on the device was more frequent with the LMA-Protector™ than with the i-gel™ (*P* = 0.033). The incidence and severity of postoperative sore throat were not different between the two groups.

**Conclusion:**

The LMA-Protector™ provided a better airway sealing effect than the i-gel™. However, it required a longer insertion time, provided a worse fiberoptic view of the vocal cords, and caused more mucosal injury compared to the i-gel™.

**Trial registration:**

ClinicalTrials.gov (NCT03078517). Registered prior to patient enrollment, Date of registration: Mar 13, 2017.

## Background

The supraglottic airway device is widely used for airway management in the anesthetic field, critical care, and emergency situations. It is also especially effective in difficult airway management. Since the introduction of the classic laryngeal mask airway (LMA), several innovative supraglottic airway devices have been developed which address such aspects as shape, quality, and function.

**Fig. 1:**
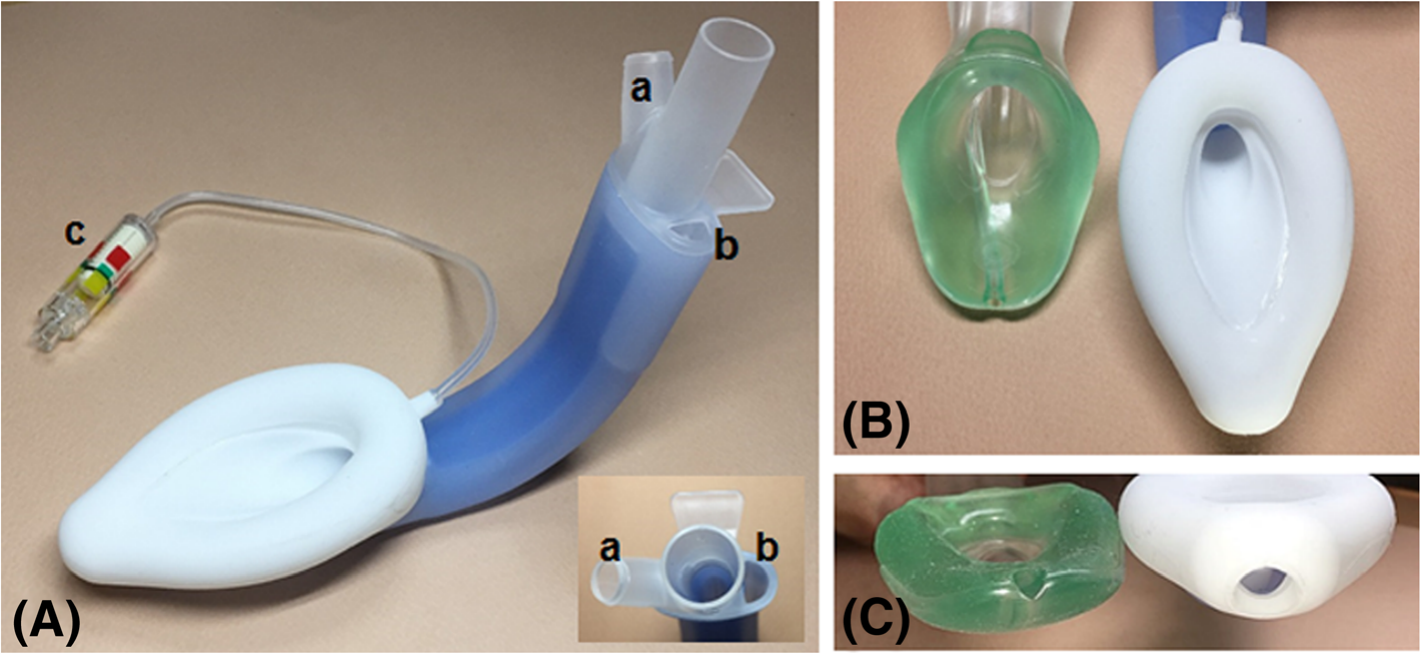
(**a**) LMA-Protector™. **a**, male suction port; **b**, female drainage port; **c**, integrated Cuff Pilot™ (**b**) Cuff of the i-gel™ and LMA-Protector™ (**c**) Distal orifice of gastric channel of the i-gel™ and LMA-Protector™. Size 4 LMA-Protector™ and i-gel™ were used for this photograph

The i-gel™ (Intersurgical Ltd., Wokingham, UK) is made of a medical-grade thermoplastic elastomer and designed to anatomically fit the perilaryngeal structures with a non-inflatable gel-like cuff that provides easier insertion and avoids compression trauma [1]. Its advantages, including easier insertion, minimal compression trauma, and sufficient airway sealing pressure have been well identified in the clinical practice [2–5]. The LMA-Protector™ (Teleflex Medical, Co. Westmeath, Ireland) is a recently developed supraglottic airway device made of medical-grade silicone which makes it more flexible and less traumatic than previous LMA devices made of polyvinylchloride. It has a fixed, curved structure for easier insertion with an inflatable airway cuff. It distinctively has two drain channels which emerge proximally as separate ports and enter a chamber behind the cuff bowl. This chamber narrows distally into the orifice located at the end of the cuff which communicates distally with the upper esophageal sphincter. Additionally, the LMA-Protector™ is available with a pilot balloon or the integrated Cuff Pilot™ that provides easier adjustment of the intracuff pressure (Fig. 1) [6].

A preliminary assessment of the LMA-Protector™ showed that it is easy to insert and provides a reliable and adequate seal [7], and a recent primary evaluation of the LMA-Protector™ reported that the LMA-Protector™ provides a high pharyngeal seal [8]. However, its performance, particularly airway sealing effect, has not been compared with other well-identified supraglottic airway devices such as the i-gel™. The i-gel™ has been widely used in the clinical practice and has been reported to show a comparable performances including airway sealing effect compared to previous LMA devices [5, 9, 10]. We hypothesized that the LMA-Protector™ would provide an improved airway seal than the i-gel™, and compared the clinical performance of the LMA-Protector™ and the i-gel™ in terms of the adequacy of airway seal, insertion time, ease and accuracy of insertion, and the incidence of postoperative sore throat in paralyzed and anesthetized patients.

## Methods

This study was approved by the Institutional Review Board of our hospital (20,170,228/26–2017-33/032), and registered at ClinicalTrials.gov (NCT03078517). After obtaining written informed consents, patients scheduled for elective surgery under general anesthesia were recruited to the study. The exclusion criteria were the presence of an upper airway anatomic variation or pathology, aspiration tendency (full stomach, history of stomach surgery, gastro-esophageal reflux, hiatal hernia), a body mass index greater than 30 kg/m^2^, a known or predicted difficult airway, surgery requiring lateral or prone position, head and neck surgery, or requirements for postoperative ventilator care.

Patients were randomly allocated to the i-gel™ or LMA-Protector™ group, using a computer-generated program (Random Allocation Software, ver. 1.0; Isfahan University of Medical Sciences, Isfahan, Iran). General anesthesia was induced with intravenous propofol 1.5–2 mg/kg, fentanyl 1–2 μg/kg and rocuronium 0.6 mg/kg. After 100 s of mask ventilation with sevoflurane in 100% oxygen, the i-gel™ or LMA-Protector™ was inserted by two board-certified staff anesthesiologists according to the manufacturer’s instruction. They inserted them alternately in each group to achieve a similar distribution for using them. The lubricated i-gel™ was inserted into the mouth and introduced along the hard palate with a continuous and gentle push in the sniffing position until resistance was felt in the hypopharynx. The LMA-Protector™ was lubricated on the posterior surface of the mask with the cuff delated. In the sniffing position, it was introduced pressing against the hard and soft palate with a circular motion until resistance was felt in the hypopharynx. The anesthesiologists had performed more than 30 insertions with the LMA-Protector™ and more than 100 insertions with the i-gel™. The size selection was made according to the manufacturer’s recommendation: for the i-gel™, a size three for patients less than 50 kg, a size four for those between 50 and 90 kg, and a size five for those over 90 kg; for the LMA Protector™, a size three for patients less than 50 kg, a size four for those between 50 and 70 kg, and a size five for those over 70 kg [1, 6].

During insertion of the device, the following manipulations were allowed: jaw thrust, adjusting insertion depth, or head extension and flexion beyond the sniffing position. If required, any maneuvers among the three were chosen at the discretion of anesthesiologists. Three attempts were allowed, and each attempt proceeded for 60 s. If the insertion was not performed within 60 s, the next attempt was made after manual ventilation. When the placement failed after three attempts, the insertion was recorded as a failure, and tracheal intubation was performed using a direct laryngoscope. The correct insertion was assessed by proper chest expansion, the presence of a square waveform on the capnogram, absence of an audible leak, and lack of gastric insufflations, as determined by epigastric auscultation. Insertion time was defined as the time from picking up the i-gel™ or LMA-Protector™ to observing the end-tidal CO_2_ waveform, and was calculated by adding the time taken for each attempt. Ease of insertion was evaluated according to the required maneuvers (jaw thrust, adjusting insertion depth, or head extension and flexion) during insertion as follows: easy for no maneuver, fair for one type of maneuver, difficult for more than one type of maneuver. The intracuff pressure of the LMA-Protector™ was set at 60 cmH_2_O, and monitored and adjusted every 30 min. The anatomic position of the devices was evaluated using a fiberoptic bronchoscope and graded on a scale of one to four as follows: four, only the vocal cords seen; three, vocal cords and posterior part of the epiglottis seen; two, vocal cords and anterior part of the epiglottis seen; one, vocal cords not seen, but adequate ventilation [11]. Airway leak pressure was determined by closing the expiratory valve of the circle system at a fresh gas flow of three L min^− 1^ and observing the airway pressure at equilibrium by auscultating the leak sound over the thyroid cartilage using a stethoscope. Airway pressure was allowed up to 40 cm H_2_O. A lubricated gastric tube was inserted through the gastric channel (size 12 Fr for i-gel™, and size 14 Fr for the LMA-Protector™). The correct placement of the gastric tube was confirmed through the injected air by auscultation of the epigastrium and aspiration of gastric content. Ease of gastric tube placement was graded as follows: one, first attempt; two, second attempt; three, impossible. Investigators who inserted the supraglottic airway device, assessed the airway leak pressure and the anatomic position of the device, and inserted the gastric tube and the observers who recorded data were not blinded to the group assignment. At the end of surgery, residual neuromuscular block was reversed by pyridostigmine and glycopyrrolate. After confirming full recovery of the spontaneous ventilation by the presence of regular and adequate trace of end-tidal CO_2_ waveform and proper chest rise without assistance, the supraglottic airway devices were removed, and the presence of blood on the device was recorded by anesthesiologists unblinded to the group assignment. The sore throat was evaluated at 1 and 24 h after surgery. A 0- to 100-mm numerical rating scale was used to evaluate the severity of sore throat (0, no pain; 100, worst pain imaginable) by investigators unaware of the group allocation. Postoperative analgesic medications was recorded for the first 24 h after surgery.

The primary outcome was the airway leak pressure. The secondary outcomes included the success rate at the first attempt, insertion time, ease and accuracy of supraglottic airway device insertion, ease of gastric tube placement, the presence of blood on the device, and incidence and severity of postoperative sore throat at 1 and 24 h after surgery.

A preliminary study was performed in 30 patients (15 per each group), and the airway leak pressure was 27.0 (6.4) cmH_2_O with the i-gel™ and 30.8 (6.6) cmH_2_O the LMA-Protector™. Based on the results of a preliminary study, a sample size calculation was performed assuming as a clinically significant difference in the airway leak pressure of 3.8 cmH_2_O between the two devices, and 50 patients per group were required at a significance level of 95% and with a power of 80%. Considering the possible dropouts, 55 patients per group were enrolled.

SPSS version 20 for Windows (IBM, Armonk, NY, USA) was used for the statistical analyses. Normality of the data was tested using the Shapiro-Wilk test. Data are expressed as mean (SD) or patient numbers (%). Student’s *t*-test or Mann-Whitney U test was used to compare the airway leak pressure, insertion time, and the severity of postoperative sore throat. The number of insertion attempts, ease of airway device, anatomic position of the device, ease of gastric tube placement, presence of blood on the device, and the occurrence of postoperative sore throat were compared using the chi-square test or Fisher’s exact test. A *P*-value < 0.05 was considered statistically significant.

## Results

**Fig. 2:**
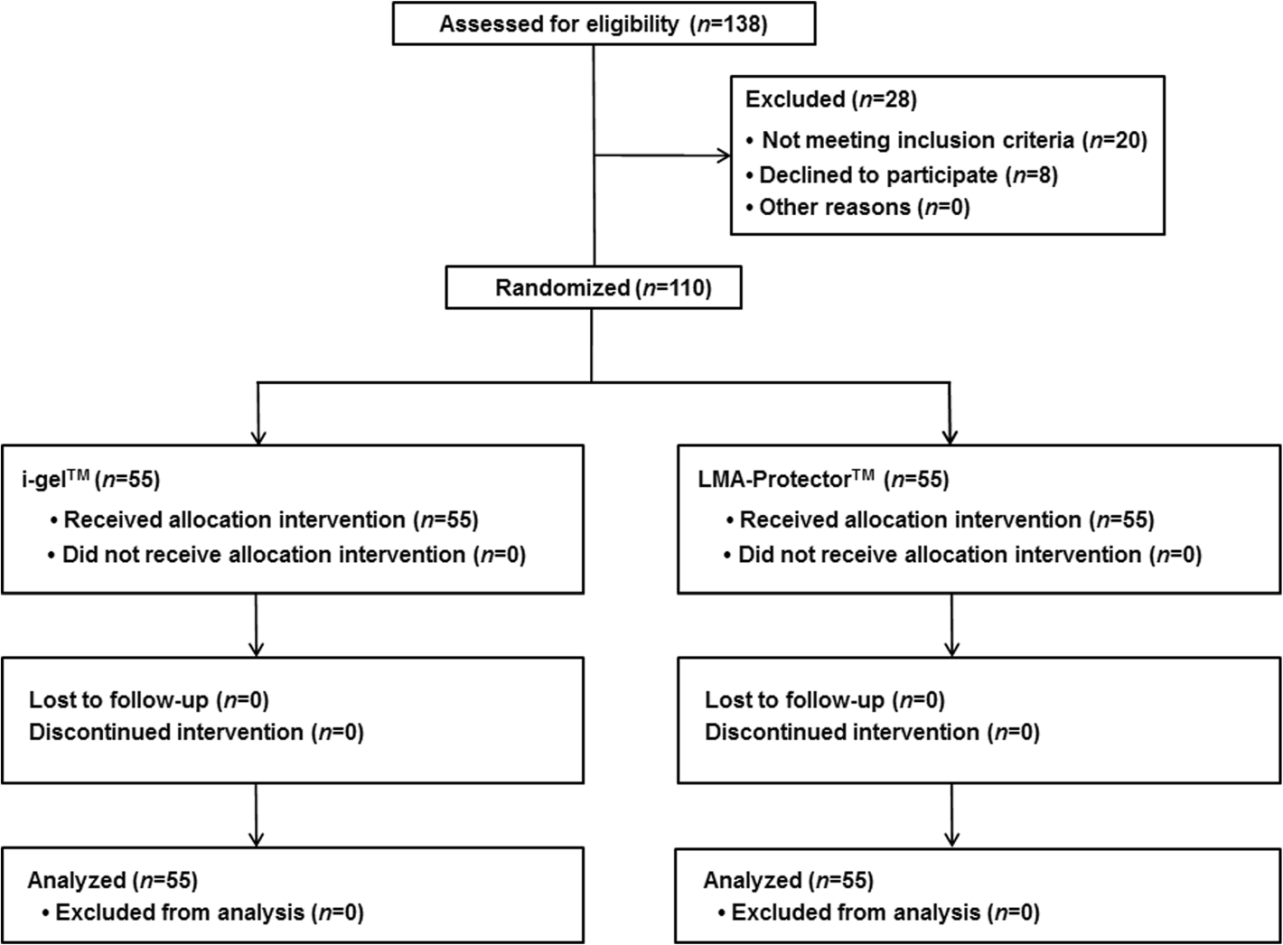
Study flowchart

A total of 138 patients were recruited from May 2017 to January 2018. Twenty patients did not fulfil the inclusion criteria, and eight patients declined to participate. One hundred and ten patients were enrolled in the study, and included in the analysis (Fig. 2). Patient characteristics, type of surgery, postoperative analgesic medications, and duration of surgery and anesthesia are presented in Table 1.

**Table 1:** Patient characteristics and surgery-related data

	i-gel™ (*n* = 55)	LMA-Protector™ (*n* = 55)
Age (years)	59 (14)	57 (18)
Gender (M/F)	28/27	27/28
Weight (kg)	65 (9)	64 (11)
Height (cm)	163 (7)	163 (8)
Postoperative analgesia		
PCA with fentanyl	32	33
Ketorolac tromethamine	7	6
Acetaminophen	4	4
Tramadol	5	5
Demorol	3	4
None	4	3
Type of surgery		
Orthopedic surgery	25	28
Urologic surgery	20	20
General surgery	10	7
Duration of surgery (min)	59 (31)	58 (38)
Duration of anesthesia (min)	90 (32)	91 (41)

Values are means (SD) or number of patients. PCA, patient-controlled analgesia

Data related to the device insertion are presented in Table 2. The airway leak pressure was significantly higher with the LMA-Protector™ than with the i-gel™ (31 [7] cmH_2_O vs. 27 [6] cmH_2_O, respectively; *P* = 0.016) Insertion time was significantly longer with the LMA-Protector™ than with the i-gel™ (27 [16] sec vs. 19 [16] sec, respectively, *P* < 0.001), but ease of insertion and the success rate on the first attempt were not different between the two groups. During the fiberoptic examination of the position of the devices, the vocal cords were totally and exclusively visualized more frequently through the i-gel™ than through the LMA-Protector™ (80% vs. 16%, respectively; *P* < 0.001). Gastric tube placement was more difficult through the LMA-Protector™ than through the i-gel™ (*P* < 0.001), and it failed in one patient through the i-gel™ and nine patients through the LMA-Protector™. Blood staining after removal of the devices was more often observed in the LMA-Protector™ group than in the i-gel™ group (24% vs. 7%, respectively; *P* = 0.033).

**Table 2:** Factors related to the device insertion

	i-gel™ (n = 55)	LMA-Protector™ (n = 55)	*P*-value
Number of insertion attempts			
one	51 (93)	50 (91)	1.000
two	4 (7)	5 (9)	
three	0 (0)	0 (0)	
Insertion time (sec)	19 (16)	27 (16)	< 0.001
Airway leak pressure (cmH_2_O)	27 (6)	31 (7)	0.016
Ease of insertion			
Easy	45 (82)	37 (67)	0.125
Fair	10 (18)	18 (33)	
Difficult	0 (0)	0 (0)	
Fibreoptic examination			
Only vocal cords	44 (80)	9 (16)	< 0.001
Vocal cords and posterior part of the epiglottis	8 (15)	28 (51)	
Vocal cords and anterior part of the epiglottis	3 (5)	11 (20)	
Vocal cords not seen, but adequate ventilation	0 (0)	7 (13)	
Ease of gastric tube insertion			
First attempt	51 (93)	27 (49)	< 0.001
Second attempt	3 (5)	19 (35)	
Impossible	1 (2)	9 (16)	
Blood staining on the device	4 (7)	13 (24)	0.033

Values are means (SD) or number of patients (%)

The incidence and severity of postoperative sore throat at 1 and 24 h were not different between the two groups (Table 3).

**Table 3:** Incidence and severity of postoperative sore throat

	i-gel™ (n = 55)	LMA-Protector™ (n = 55)	*P* − value
Incidence			
1 h after surgery	11 (20)	16 (29)	0.376
24 h after surgery	8 (15)	9 (16)	1.000
Severity			
1 h after surgery	4 (11)	9 (17)	0.168
24 h after surgery	3 (7)	3 (8)	0.794

Values are means (SD) or number of patients (%). Severity of postoperative sore throat was assessed using a 0- to 100-mm numerical rating scale (0, no pain; 100, worst pain imaginable)

## Discussion

This study showed that the LMA-Protector™ provides a better airway sealing effect than the i-gel™, however, the LMA-Protector™ required a longer insertion time, provided a worse fiberoptic view of the vocal cords, and caused more mucosal injury.

In the present study, the mean airway leak pressure was higher with the LMA-Protector™ (31 cmH_2_O) than with the i-gel™ (27 cmH_2_O), consistent with the results of the previous studies showing values of 23–29 cmH_2_O for the i-gel™ [5, 9, 10, 12]. The oropharyngeal airway seal, quantified by the airway leak pressure, is essential for the prevention of aspiration and ventilator efficiency. Higher airway leak pressure results from the closer contact between the cuff and the adjacent soft tissues. The LMA-Protector™ cuff, made of medical-grade silicone, may provide a more individualized fit in the pharynx and hypopharynx. According to a preliminary evaluation, the median pharyngeal seal pressure of the LMA-Protector™ was 34 cmH_2_O [8]. In another preliminary assessment in non-paralyzed female patients with the LMA-Protector™ size three [7], median oropharyngeal leak pressure was 25.2 cmH_2_O. In the present study, we used the different sized devices (size three, four or five) according to the manufacturer’s recommendation based on the patient’s weight in paralyzed males and females. Several factors such as the use of neuromuscular blockade and the size of the device, may have affected the airway leak pressure.

In the present study, the mean airway leak pressure was slightly higher with the LMA-Protector™ than with the i-gel™, which might be clinically insignificant. However, this result should not be ignored because it may also suggest that the LMA-Protector™ can be a choice in some clinical situations where a higher airway leak pressure is required such as laparoscopic surgery, although it was not evaluated in this study.

The success rate of the device insertion at the first attempt (91% vs. 93%, LMA-Protector™ vs. i-gel™, respectively), and ease of insertion were not different between the two devices, but the insertion time was longer with the LMA-Protector™ than with the i-gel™. The i-gel™ has a non-inflatable cuff, whereas the LMA-Protector™ has a longer and larger inflatable cuff, therefore, it might take more time to introduce the larger cuff into the oropharyngeal space and inflate it. Moreover, anesthesiologists had more familiarity with the i-gel™ than the LMA-Protector™, which may influence insertion time.

The i-gel™ and LMA-Protector™ can be used as an intubation conduit, and proper alignment of the ventilation pathway with the vocal cords is crucial for successful tracheal intubation. In the present study, ventilation was adequate in all patients, but the i-gel™ had a better fiberoptic view of the glottis with less epiglottic down-folding than the LMA-Protector™. This finding was consistent with previous studies in which the i-gel™ provided an acceptable fiberoptic view of the vocal cords in more than 80% of subjects [5, 10]. The i-gel™ has an epiglottic rest preventing the epiglottis from down-folding or obstructing the distal opening the airway [1], whereas the LMA-Protector™ has no component to prevent epiglottic down-folding, such as the epiglottic elevating bar in the LMA-Fastrach™ [13, 14].

The i-gel™ has one gastric channel, similar to other pre-existing supraglottic airway devices. The newly developed LMA-Protector™ distinctively contains two gastric channels, a suction port and a drainage port, which emerge proximally as separate ports, entered a chamber located behind the cuff bowl, and communicates distally with the upper esophageal sphincter (Fig. 1). The gastric fluid can be removed by attaching suction to the suction port or by inserting a gastric tube through the drainage port to the stomach. The internal volume of the drainage pathway in the LMA-Protector™ (31 mL for size three; 41 mL for size four; 42 mL for size five) [13] is much larger than that of the i-gel™. Thus, the LMA-Protector™ may be more efficient at reducing the risk of pulmonary aspiration of gastric contents. Nevertheless, in the present study, the gastric tube insertion was more difficult through the LMA-Protector™ than through the i-gel™ despite of adequate ventilation in all patients. We failed to insert the gastric tube in nine patients through the LMA-Protector™ and one patient through the i-gel™. This finding might be associated with the size of the gastric tube used with the LMA-Protector™. According to the instructions for use of each device, the recommended maximum size of the gastric tube was 12 or 14 Fr for the i-gel™ (12 Fr for sizes three and four; 14 Fr for size five) and 16 or 18 Fr for the LMA-Protector™ (16 Fr for size three; 18 Fr for sizes four and five) [1, 6]. In the present study, we used the gastric tube size 12 Fr for the i-gel™ and size 14 Fr for the LMA-Protector™. The selection of the 14 Fr gastric tube for the LMA-Protector™ was based on a preliminary study using the LMA-Protector™ size three in female patients in which the 14 Fr gastric tube was successfully inserted in 24 of 25 patients [7]. In this study, we inserted the 14 Fr gastric tube through the size three, four or five LMA-Protector™, and considered that it was difficult to introduce the relatively thin and flexible gastric tube within the large drainage pathway. Thus, the use of a larger sized gastric tube might facilitate the passage through the gastric channel of the LMA-Protector™ although it was not evaluated in this study. Moreover, the LMA-Protector™ has a large gastric pathway, which may provide a potential advantage at risk of pulmonary aspiration. Thus, a further study is required regarding the protective effect of the LMA-Protector™ against pulmonary aspiration.

In the present study, blood staining indicative of mucosal injury was more frequent with the LMA-Protector™ (24%) than with the i-gel™ (7%). In some previous studies, blood staining was observed in 0–13% of patients with the i-gel™ [9, 15–17]. The i-gel™ has a non-inflatable cuff made of a soft, gel-like medical-grade thermoplastic elastomer, potentially reducing the oropharyngeal tissue injury [18]. Although the LMA-Protector™ is made of flexible medical-grade silicone, it has a strongly tapered leading tip and a longer and larger inflatable cuff compared to the i-gel™, which may cause more mucosal injury. The incidence and severity of postoperative sore throat, however, were not different between the two devices.

This study had several limitations. First, the investigators who inserted the supraglottic airway device were not blinded to the group assignment due to the nature of the study. They followed the standardized and detailed study protocol. The investigators that evaluated postoperative sore throat, and all patients were blinded to the group allocation. Yet, there is still the potential for bias. Second, the investigators who inserted the supraglottic airway device had more experience with the i-gel™ (more than 100 insertions) than with the LMA-Protector™ (more than 30 insertions). They did have experience with more than 50 insertions of the LMA-Supreme™ which has a similar insertion method to the LMA-Protector™. However, a conscious (or unconscious) bias against the newer device, LMA-Protector™, might affect the results. Furthermore, the difference in experience with the two devices, especially less experience with the newer device, may be a possible source of bias. Third, this study was performed in anesthetized and paralyzed patients with normal airways. Thus, our results cannot be generalized to non-paralyzed patients, patients during spontaneous ventilation, and patients with difficult airways. Fourth, this study was conducted in patients with a mean body mass index of 24 kg/m^2^, not in obese patients, and our results may not be applicable to obese patients.

## Conclusions

LMA-Protector™ provided a higher airway sealing effect, but provided a worse fiberoptic view of the vocal cords, and caused more mucosal injury compared to the i-gel™. The gastric tube placement was more difficult with the LMA-Protector™ than with the i-gel™, however this may be related to the gastric tube size used in our study. A larger sized gastric tube, within the range of recommended sizes, might better facilitate the passage through the gastric channel of the LMA-Protector™. Moreover, the LMA-Protector™ has a distinctively large gastric pathway, and a further study is required regarding the protective effect of the LMA-Protector™ against pulmonary aspiration.

## Data Availability

The dataset supporting the conclusions of this article is included in this article and its supplemental information file.

## Abbreviations

LMA: laryngeal mask airway
PCA: patient-controlled analgesia
